# Theranostic Advances of Bionanomaterials against Gestational Diabetes Mellitus: A Preliminary Review

**DOI:** 10.3390/jfb12040054

**Published:** 2021-09-28

**Authors:** Mahmood Barani, Saman Sargazi, Vahideh Mohammadzadeh, Abbas Rahdar, Sadanand Pandey, Niraj Kumar Jha, Piyush Kumar Gupta, Vijay Kumar Thakur

**Affiliations:** 1 Medical Mycology and Bacteriology Research Center, Kerman University of Medical Sciences, Kerman 7616913555, Iran; Mahmoodbarani7@gmail.com; 2 Cellular and Molecular Research Center, Research Institute of Cellular and Molecular Sciences in Infectious Diseases, Zahedan University of Medical Sciences, Zahedan 9816743463, Iran; sgz.biomed@gmail.com; 3 Department of Pharmaceutical Nanotechnology, School of Pharmacy, Mashhad University of Medical Science, Mashhad 1313199137, Iran; MohammadzadehV971@mums.ac.ir; 4 Department of Physics, Faculty of Science, University of Zabol, Zabol 53898615, Iran; 5 Department of Chemistry, College of Natural Science, Yeungnam University, 280 Daehak-ro, Gyeongsan 38541, Gyeongbuk, Korea; sadanand.au@gmail.com; 6 Department of Biotechnology, School of Engineering and Technology, Sharda University, Greater Noida 201310, India; nirajkumarjha2011@gmail.com; 7 Department of Life Sciences, School of Basic Sciences and Research, Sharda University, Greater Noida 201310, India; 8 Biorefining and Advanced Materials Research Centre, SRUC, Edinburgh EH9 3JG, UK; 9 Department of Mechanical Engineering, School of Engineering, Shiv Nadar University, Noida 201314, India; 10 School of Engineering, University of Petroleum & Energy Studies (UPES), Dehradun 248007, India

**Keywords:** gestational diabetes mellitus, diagnosis, treatment, nanomaterials, nanotechnology

## Abstract

Gestational diabetes mellitus (GDM) is the most frequent complication during pregnancy. This complex disease is characterized by glucose intolerance and consequent hyperglycemia that begins or is first diagnosed in pregnancy, and affects almost 7% of pregnant women. Previous reports have shown that GDM is associated with increased pregnancy complications and might cause abnormal fetal development. At present, treatments are not suitable for the prevention and management of these patients. As an alternative therapeutic opportunity and a leading scientific technique, nanotechnology has helped enlighten the health of these affected women. Theranostic nanomaterials with unique properties and small sizes (at least <100 nm in one of their dimensions) have been recently engineered for clinics and pharmaceutics. Reducing materials to the nanoscale has successfully changed their properties and enabled them to uniquely interact with cell biomolecules. Several biosensing methods have been developed to monitor glucose levels in GDM patients. Moreover, cerium oxide nanoparticles (NPs), selenium NPs, polymeric NPs, and drug-loaded NPs loaded with therapeutic agents have been used for GDM treatment. Still, there are some challenges associated with the detection limits and toxicity of such nanomaterials. This preliminary review covers the aspects from a fast-developing field to generating nanomaterials and their applications in GDM diagnosis and treatment.

## 1. Introduction

Diabetes mellitus (DM) has reached epidemic proportions and is a leading cause of death worldwide (despite the decades of clinical studies and trials of novel therapeutic strategies) [1]. Because of inaccuracy and insufficiency of data for monitoring DM patients, particularly in developing countries, there is a significant gap in comprehending the burden nationally and globally [2]. The World Health Organization (WHO) estimated that the prevalence of DM in adults would rise to 300 million cases by 2025 [3]. This number includes patients with GDM and should alert healthcare providers to concentrate on preventive actions before childbirth.

GDM, defined as any level of glucose intolerance with onset or first recognition during pregnancy, affects about 7% of all pregnancies worldwide and poses life-threatening short- and long-term risks for the mother and the baby [4,5,6]. Inauspiciously, these health consequences emerge at the maternal glucose values [7]. GDM is characterized by the failure of pancreatic β-cells to respond appropriately to the insulin requirements during gestation, which leads to hyperglycemia [8]. Obesity, family history of DM, age, and ethnicity are among the main factors that may enhance the risk of GDM [9]. It has been well established that most women with GDM return to the normoglycemic state soon after childbirth. Until now, the consequences of GDM have extended beyond the pregnancy, with affected women conferring a seven-times increased risk of developing type 2 diabetes mellitus (T2DM) compared with women who maintained normoglycemic during maternity [10].

For screening GDM, all pregnant women should undergo oral glucose testing with 50-g glucose at 24 to 28 weeks of gestation. If glucose tolerance is impaired, a subsequent glucose tolerance test should be carried out to diagnose GDM [11]. At present, GDM diagnosis is made by a 75-g or 100-g oral glucose tolerance test [12]. Still, this test has limitations, and a single test cannot confirm the GDM diagnosis [11]. Regarding GDM treatment, various efforts have been made to reverse hyperglycemia and decrease the risk of the related adverse pregnancy outcomes [8]. Furthermore, lifestyle interventions, pharmacological therapies (i.e., insulin therapy and administration of metformin or glibenclamide), and postnatal managements present several therapeutic options associated with the enhanced glycemic control for both the mother and the child [8,13,14]. With the increasing prevalence of T2DM, specifically in the deprived areas, the precise diagnosis of GDM is now considered an encouraging opportunity for the intervention to alleviate the burden of T2DM [15]. Accordingly, it seems imperative to develop new theranostic platforms for the accurate diagnosis of this condition.

Recently, the advances in nanomedicine have prompted the designing of favorable therapeutic modalities for various applications [16,17,18,19]. Furthermore, nanomedicine has influenced these efforts by increasing the surface area of the biosensors, enhancing the catalytic properties of the electrodes, and creating nanoscale sensors for a wide range of theranostic purposes [20]. Nanomaterials, such as NPs [21,22,23,24], block-copolymer micelles [25], nanocapsules [26], nanocages [27], and nanocarriers (i.e., nanoliposomes [28]), nanocomposites [29], and nanohydrogels [30]) with well-controlled properties have emerged for monitoring the blood glucose levels as well as therapy and care of DM and/or GDM patients. These nanomaterials mostly assisted in the direct measurement of glucose in serum or substantially improved the glucose sensor function. Moreover, they acted as the newly developed drug delivery systems (DDSs) to achieve active targeting [31]. The small-targeted DDSs can ameliorate the severity of DM in patients and promotes the growth and development of pancreatic β-cells via inducing the Wnt signaling pathway, activating the autophagic target points, inhibiting inflammasome, and triggering other molecular pathways [32]. Various nanosensors, including engineering periplasmic ligand-binding proteins [33], acetone nanosensors [34], near-infrared optical nanosensors [35], copolymer-based fluorescence nanosensors [36], graphene field-effect transistor nanosensors [37], silver nanoparticle-modified nanosensors [38], and other biological nanosensors have been designed as non-invasive diabetes sensing technologies for the sensitive detection of glucose in the affected patients [39,40]. For the diagnosis of GDM, intensive development on biomarker sensing is currently being conducted in advanced fields with the help of such nanomaterials.

Several reports are currently available on the therapeutic effects of nanomaterials against GDM in both in vitro and in vivo models. Most of these nanomaterials have offered to increase the stability and therapeutic effects of anti-GDM agents. For instance, Du et al. found that the chitosan encapsulated nano-resveratrol could diminish the level of interleukin 6 (IL-6), a pro-inflammatory factor, and reduce the markers of endoplasmic reticulum stress in streptozotocin-induced GDM rats [41]. In another study, Cheng and colleagues fabricated the biogenic polyacrylic NPs for GDM therapy and tested these NPs in a rat model.

Available review articles primarily discussed the role of nanotechnology in the treatment and diagnosis of diabetes mellitus. To the best of our knowledge, there are no comprehensive reports on theranostic advances of nanomaterials against GDM. Hence, this preliminary review discusses the recent findings and provides an empirical perspective on the implications of nanomedicine for the diagnosis and cure of GDM in affected women. In the end, we will also discuss the existing challenges and limitations of nanotechnology-based approaches in this field.

## 2. Diagnosis of GDM

### 2.1. Potential Biomarkers

**Figure 1:**
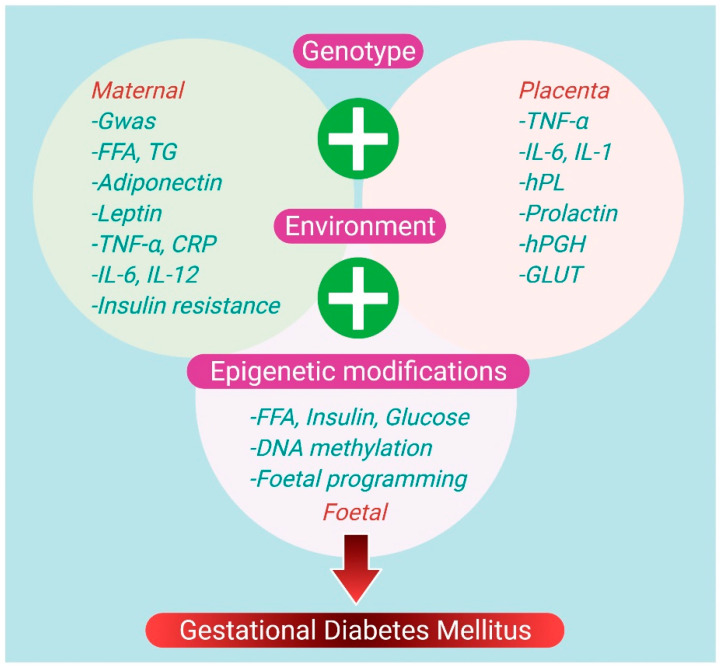
Potential biomarkers and recognized pathophysiologic mechanisms in GDM Abbreviations: free fatty acids (FFA), genome-wide association study (GWAS), interleukin-6 (IL-6), triglycerides (TG), human placental growth hormone (hPGH), glucose transporter (GLUT), human placental growth hormone (hPGH), tumor necrosis factor-alpha (TNF-α), C-reactive protein (CRP), interleukin-12 (IL-12), interleukin-1 (IL-1), and human placental lactogen (hPL).

The specific biomarkers can target the treatment and potentially reduce the incidence of GDM in women at a high risk of developing it. Overweight/obesity, age, race, and family-related diabetes are risk factors of GDM, but the lack of specificity limits the GDM diagnosis [42]. The simultaneous use of GDM predictive models can improve treatment effectiveness for women at risk of developing GDM. The pathophysiologic causes of GDM include chronic inflammation, impaired placental function, and insulin resistance, which are reflected as predictive and diagnostic biomarkers. Furthermore, the significance of epigenetic modifications in GDM pathogenesis highlights an intricate relationship between the environmental and genetic variables, thus improving the risk prediction of GDM disease [43]. In GDM research, numerous differentially expressed biomarkers have been explored, offering a better understanding of the intricacies of GDM pathophysiology and functioning as prospective diagnostic indicators. The essential biomarkers for GDM detection are adipokines (leptin, tumor necrosis factor (TNF), interleukin 6 (IL-6), etc.), glycoproteins (afamin, CD59, sex-hormone binding protein (SHBG)), pregnancy-associated plasma protein-A (PAPP-A), C-reactive protein (CRP), and retinol-binding protein 4 (RBP4) (Figure 1) [44,45]. To bring the screening and diagnosis of GDM disease into the 21st century, several ongoing research efforts will continue to develop more effective and accurate biomarkers. Moreover, nanotechnology can help us better understand the GDM pathophysiology and improve its diagnosis at an earlier stage.

### 2.2. Role of Nanotechnology in GDM Diagnosis

Many women who are diagnosed with GDM may have had undiagnosed hyperglycemia before pregnancy. Screening for hyperglycemia should ideally occur as part of well-resourced and well-organized preconception care in high-prevalence countries. However, this strategy has limitations because only about 40% of pregnancies across the globe are managed [46]. We cannot say for sure that testing in early pregnancy predicts GDM because there is no preconception testing. On the other hand, early testing provides an opportunity to identify those women who are likely to have pre-existing glucose metabolism problems [47]. Nanotechnology has made rapid advancements that can be used to solve these problems. The effectiveness of high-performance diagnostic employing relevant biomarkers in diagnosing GDM has been demonstrated in many studies [48,49].

**Figure 2:**
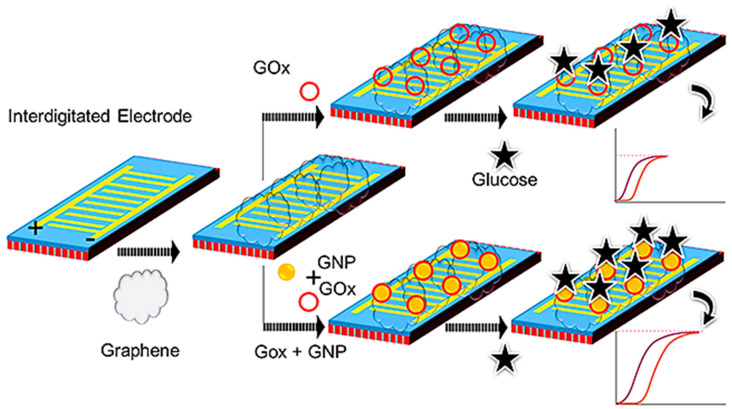
Schematic representation of GOx-Au NPs on graphene and GOx on graphene for dielectric sensing. Abbreviations: gold NP.s (Au NPs) and glucose oxidase (GOx) [50].

Because of the significant number of pregnant women afflicted, it should be essential to assess the level of glucose during pregnancy, and of course, a continuous assessing platform is needed. Ge et al. employed graphene-GOx-Au NPs and graphene-GOx modified IDE sensing surfaces to investigate the quantity of glucose interaction (Figure 2). The sensitivity of this method was determined to be 0.06 mg/mL, and GOx was coupled with Au NPs to improve its detection. The Au NPs-GOx had a higher level of current changes and a two-fold increase in sensitivity detection (from 0.06 increased to 0.03 mg/mL) at all of the glucose concentrations examined. The specificity, repeatability, and increased sensitivity detections of the above IDE sensing system demonstrated its good performance. Furthermore, the LOD was estimated to be between 0.02 and 0.03 mg/mL using linear regression analysis [50]. This study demonstrated the potential strategy with nanocomposite for diagnosing gestational diabetes mellitus.

In a similar study, with the help of Au nanorod (AuNR) that conjugated to glucose oxidase (GOx) on an interdigitated electrode sensor, Zheng et al. detected glucose at a LOD of 0.06 mg/mL. In the absence of AuNR, GOx indicated that the LOD of glucose was about 0.25 mg/mL. Furthermore, the reactions of all glucose concentrations have acquired larger levels of current with GOx-GNR compared to the baseline. The specificity evaluation revealed that the glucose only interacts with GOx-GNR and effectively discriminates other sugars. This kind of monitoring can be exploited to determine and continuously manage glucose levels during pregnancy and postpartum [51]. This method of detection is useful to diagnose and continuously monitor the glucose level during the pregnancy period.

In another study, Chen et al. developed a silica-alumina (Si-Al)-modified capacitive non-Faradaic glucose biosensor for GDM monitoring. Through amine-modification, GOx (as a probe) was attached to the surface of the Si-Al electrode. When GOx binds to glucose, the Si-Al (with the size of 50 nm) modified electrode surface boosted the current flow. The glucose concentrations were raised to boost capacitance values. A mean capacitance value was plotted on the linear range between 0.03 and 1 mg/mL, and the LOD was found to be 0.03 mg/mL (R^2^ = 0.9782). Furthermore, a biofouling experiment with galactose and fructose did not raise the capacitance, demonstrating that GDM requires specialized glucose monitoring [52]. This Si-Al-modified capacitance sensor detects a lower level of glucose presence and helps in monitoring gestational diabetes.

Pandey et al. also proposed an electrochemical sensor based on dual imprinted polymer-based flexible and nanocubes to simultaneously monitor multi diabetes indicators, including non-glycated and glycated hemoglobin. For this purpose, electropolymerization was used to deposit poly-rhodamine b nanocubes and dual molecularly imprinted poly-aminophenyl boronic acid on the surface of the electrode (aluminum foil and carbon paste). The selective targeting of glycated hemoglobin and hemoglobin in their complementary positions was due to cis–diol interactions and non-covalent bondings with poly-aminophenyl boronic acid and poly-rhodamine b. Electrochemical tests showed that the suggested flexible sensor could electrochemically catalyze both hemoglobin and glycated hemoglobin redox reactions simultaneously and that its electrochemical responsiveness remained intact after 450 bends. The LOD of hemoglobin and glycated hemoglobin were reported to be as low as 0.08 and 0.09 ng/mL under optimal circumstances. Blood samples from diabetic and healthy pregnant women were used to test the dependability of the proposed flexible sensor using a standard chromatographic approach [53].

As previously stated, hemoglobin A1c (HbA1c) and glucose are the gold biomarkers currently applied for GDM diagnosis. However, HbA1c represents 2–3 months of glycemic information and is too rare for monitoring the clinical impact of GDM Furthermore, glucose offers numerous daily measurements that are arguably unnecessary for mild to moderate GDM, and frequently result in patient non-compliance [54]. As a result, an alternative biomarker is needed to detect the glycemic state of GDM patients effectively. The most common protein in serum albumin or blood is glycated non-enzymatically in the bloodstream. It can be utilized as an intermediate biomarker because of its half-life of 21 days [55]. Glycation of albumin usually is between 10 and 16%, but it is substantially higher in diabetes patients, between 16 and 40%. In this light, Belsare et al. designed a diagnosis device with a point-of-care (POC) manner to determine glycated albumin (GA) as a percent of total serum albumin. Briefly, an aptamer approach using Au NPs was utilized to obtain colorimetric data in a dipstick paper fluidic test to quantify percent glycated albumin. Glycated and un-glycated serum albumin were assessed in their physiological concentration ranges (500 to 750 μM for un-glycated serum albumin and 50 to 300 μM for glycated albumin), with a LOD of 21 and 6.5 μM for un-glycated and glycated serum albumin. The use of aptamers as recognition elements, instead of commonly used antibodies, providing not only the required sensitivity, specificity, and dynamic range but also has the added advantage of being stable at room temperature for an extended period, providing the potential for these dipstick tests to be used for GDM monitoring at the point-of-care (POC) [56].

**Figure 3:**
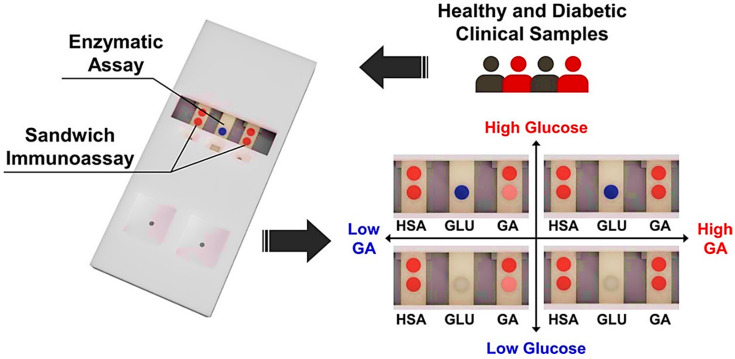
Representation of the sandwich immunoassay sensor in the detection of glycated albumin (GA) glucose (GLU) and human serum albumin (HSA) for diagnosis of GDM [57].

Moreover, in a similar study, using a lateral flow experiment and Au NPs, Ki et al. designed a sensor that can identify the glycation ratios of human blood albumin and glucose levels at the same time. A spiked glucose solution, total human serum albumin, and glycated albumin were tested simultaneously using particular enzyme reactions and immunoassays. Clinical serum samples from healthy persons and diabetic patients were tested to test the performance of the proposed sensor. Glucose levels of the samples and glycation ratios were shown to be reasonably correlated. The glucose level and glycation ratio assessments had R-squared values of 0.932 and 0.930, respectively. The sensor’s average recognition recoveries for glycation ratio and glucose were 98.32% and 85.80%, respectively. Based on the outcomes of the present study, they proposed that this novel platform could be utilized for the simultaneous detection of glucose and glycation ratios to diagnose and monitor diabetes mellitus [57]. Representation of the sandwich immunoassay sensor in the detection of glycated albumin (GA) glucose (GLU), and human serum albumin (HSA) for diagnosis of GDM is demonstrated in Figure 3.

On the other hand, elucidating mechanisms of GDM by integrating proteomics and other omics technologies have recently gained considerable attention [58]. In this respect, there is a report on detecting the sequence of some peptides that are differentially expressed between healthy women and GDM cases using a non-liquid chromatography-electrospray ionization-tandem mass spectrometry (nano-LC/E.S.I.–MS/MS) system and a mass spectrometer [59]. This method allows sensitive detection of derivatized peptides, as GDM biomarkers, at attomole levels [60].

## 3. Nanotechnology for Treatment of GDM

As discussed earlier, GDM is a condition of glucose intolerance, in which a person who does not have diabetes will experience hyperglycemia during pregnancy. Therefore, the onset and first diagnosis of this diabetes occur during pregnancy. Risk factors include being overweight, a history of previous GDM, a family history of type 2 diabetes, and polycystic ovary syndrome. A blood test is used to diagnosis this type of diabetes [61,62]. GDM can occur due to insulin resistance or decreased insulin production. It also increases the incidence of congenital malformations in the fetus. According to research, mitochondrial damage and oxidative stress are the most influential factors in diabetic fetuses [21].

### 3.1. Use of Metallic NPs for Treatment of GDM

Cerium is the second element in the lanthanide series in the periodic table. It is one of the rare elements of the planet, often showing a +3-oxidation state, but is also stable in the +4 state. Cerium has no biological role in humans and is not very toxic [63]. Cerium oxide (CeO_2_), in combination with oxygen in an NP. formulation, forms an alloy crystal structure that exhibits profound antioxidant properties [64,65]. CeO_2_ NPs are potential new drugs for oxidative disorders that overcome the weaknesses of previous treatments and ischemic brain damage [21,66]. In a study by Vafaei-Pour et al. in diabetic rats, they used nanoceria as an antioxidant to improve fetal diabetes treatment. Diabetes was induced by a dose of streptozotocin and blood glucose levels were calculated on the 0, 5th, 10th, and 15th day of pregnancy. Diabetes was confirmed when the blood glucose concentration reached more than 200 mg/dL. Oxidative stress, pathological parameters, abortion, and live embryos were assessed. Histological studies showed that diabetes causes abortion. Nanoceria treatment inhibited embryonic oxidative stress as well as pathological changes in diabetic rats. Because diabetes has a teratogenic nature, nanocrystals help treat a diabetic fetus through their antioxidant effects. Therefore, early diagnosis of GDM and administration of antioxidants can reduce these complications [21]. In another study, Vafaei-pour et al. investigated the protective effect of ceria NPs in preventing mitochondrial damage due to GDM After induction of diabetes by streptozotocin and reaching blood glucose above 200 mg/dL on the 16th day of gestation, the embryo was isolated, and the mitochondria were purified by centrifugation. Markers related to mitochondrial damage and oxidative stress were then analyzed. The results showed that treatment with nanoceria at a dose of 60 mg/kg significantly prevented the development of oxidative stress and mitochondrial toxicity (*p* < 0.05) [67]. The defensive effect of CeO_2_ NPs in diabetic mice was investigated. CeO_2_ NPs enhanced the morphological abnormalities of dorsal root ganglion neurons (DRG). Administration of CeO_2_ NPs for 8 weeks significantly reduced the ADP/ATP level in diabetic rats compared to non-diabetic rats (*p* < 0.001). This study showed that the effect of diabetes was repressed by CeO_2_ NPs [68].

Selenium (Se) is present in plants and is a rare element. Selenium deficiency in the body causes various diseases, including diabetes. This element has antioxidant properties, and Se NPs can inhibit tissue oxidation by inhibiting numerous peroxides, protecting lipids and cellular macromolecules from oxidative damage to membranes, growing glutathione peroxidase levels, then thyroxine reductase [69,70]. In a study of T2DM mice, Hanaa et al. found that selenium-containing liposomes maintain β-cell integrity, enhance insulin excretion, lower glucose levels, restore the equilibrium of oxidative, antioxidant production, and reduce pancreatitis; therefore, they have antidiabetic properties [71]. Hassan et al. examined the effect of Se NPs and their therapeutic effects on puppies of mothers with GDM, after administration of 5 mg/kg body weight twice a week for one month. Blood-, pancreas-, and kidney-sacrificed puppies were then biochemically analyzed, and tissues were studied. The results showed that puppies of diabetic mothers treated with synthesized NPs displayed good redox parameters (reduction of glutathione and malondialdehyde in tissue samples). The current findings suggested that the Se NPs could counteract the diabetes-related complications in offspring by reorganizing the cellular redox state. Therefore, the present study shows that Se NPs acted protectively in diabetic mothers containing GDM and did not allow their infants to pass [72].

In 2021, Wang et al. designed an antidiabetic drug delivery device by mimicking pancreatic cells. In this study, hollow mesoporous silica nanoparticles with dual-responsive copolymer coatings were used for subcutaneous delivery of glucose. The dual-response glucose drug delivery system involves a combination of pH and H_2_O_2_ reacting with a bonded copolymer of hollow mesoporous silica nanoparticles (HMSNs), with a microneedle (MN) patch array. Poly (4-(4,4,5,5-tetramethyl-1,3,2-dioxaborolan-2-yl) benzyl acrylate) -b-poly (2-(dimethylamino) ethyl methacrylate) (PPBEM-b-PDM)—the polymer holds the gate and prevents the drug from secreting from the HMSN cavity at the normoglycemic level. Moreover, due to the chemical change of the H_2_O_2_-sensitive PPBEM block and acid-responsive PDM block on H_2_O_2_ and pH stimuli, the drug release rate increases significantly. The combination of antidiabetic and glucose oxidase in HMSNs coated with stimulant polymers results in forming a glucose-mediated MN device after deposition of drug-laden nanoparticles to MN Laboratory and in vivo results showed that the MN device has the property of releasing the drug with glucose adjustment, which has a rapid release of the drug at the level of high blood sugar, but the release of the drug at the normoglycemic level is delayed. Therefore, such a drug delivery system can be very effective in treating diabetes [73].

### 3.2. Use of Polymeric NPs for GDM Treatment

**Figure 4:**
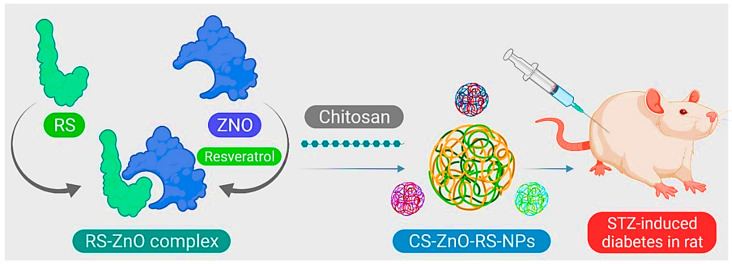
Schematic of CS-ZnO-RS nanoparticle preparation for GDM treatment.

Chitosan (CS) is the second richest polysaccharide in nature next to cellulose. An amino polysaccharide is a linear product gained through alkaline acetylation of chitin (found in the exoskeleton of certain crustaceans for example shrimp, crabs). Chitosan is biocompatible, degradable, and non-toxic. It can chelate with metal ions. As a result of its cationic and high charge density, CS has various applications in preparing materials, such as flocculants, coagulants, food additives, and weight loss/pharmaceutical formulations [74]. In the study by Du et al., zinc oxide (ZnO)-resveratrol (RS) was encapsulated with CS, and CS-ZnO-RS NPs were synthesized (Figure 4). Characterization of the NPs by electron microscopy, besides particle analysis, proved that the synthesized CS-ZnO-RS NPs were spherical in shape and had an average size of 38 nm. Moreover, the therapeutic properties of these NPs on GDM were investigated. The results showed that CS-ZnO-RS NPs were able to deliver resveratrol by reducing the side effects and increasing bioavailability. These NPs significantly reduced blood glucose levels, and fat levels in mice with GDM. CS-ZnO-RS NPs at a concentration of 500 μg/mL inhibited α-glucosidase (77.32%) and α-amylase (78.4%). It also reduced the levels of inflammatory agents (IL-6 and MCP-1) in addition to endoplasmic reticulum stress (GRP78, p-IRE1α, p-eIF2α, and p-PERK) [41].

**Figure 5:**
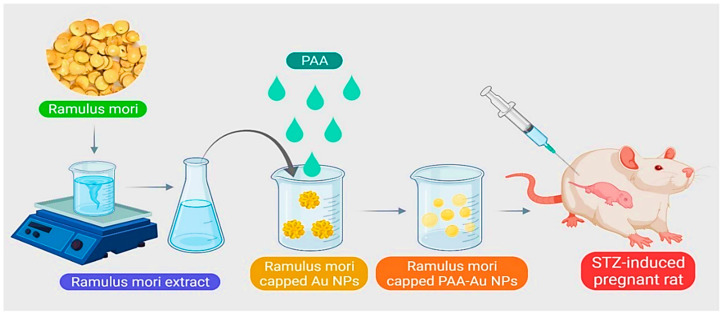
Schematic representation for synthesizing polyacrylic gold NPs (PAA-Au NPs) using chemical polymerization to treat GDM.

Presently, for the treatment of GDM, it is difficult to deliver drugs accurately and appropriately to the intended treatment site. Uses of gold NPs include use in the treatment of diabetes mellitus, insulin transport, anti-diabetes, and as carriers for delivering various drugs. A research study by Cheng et al. proposed a new method for releasing and producing a diabetic drug. Using the green synthesis method, *Ramulus mori* methanolic extract (RME) was loaded on polyacrylic gold NPs (PAA-Au) using chemical polymerization and examined for GDM treatment (Figure 5). FT-IR results showed the formation of Au-PAA-NPs extract. The results of microscopic observations in diabetic mother rats showed normal variations in liver cell layers. The rat liver received Au NPs and caused significant improvement in liver tissue. Biochemical tests also showed that the use of Au-PAA-NPs improves changes in serum glucose levels in the mother. The present study showed that AuNPs are active in contrast to diabetes. Therefore, it has introduced a new method for treating GDM [75].

In another study by Yan et al., *Murraya koenigii* extract (*M. koenigii*) and Au-PLGA nanoformulation were synthesized. GDM in rats was induced by streptozotocin (STZ). As a result of treatment with *M. koenigii* leaf extract with Au-PLGA nanoformulation, serum levels of lipids and glucose were significantly increased. In pancreas and liver tissue, levels of antioxidant enzymes, due to GDM, were significantly reduced, and levels of cell-strengthening compounds in the pancreas and liver tissue in diabetic rats were the same as in control. *M. koenigii* leaf extract, rich in antioxidants, is very effective and can protect cells against chemicals, suppress oxidative blood pressure and insulin and, thus, increase the blood glucose level of GDM in rats [76].

## 4. Challenges in GDM Diagnosis by Use of Nanosensors

As mentioned above, glucose nanosensors (i.e., NPs, nanotubes, and nanocomposites) were incorporated into implantable devices to function as continuous glucose monitors (C.G.M.s). Although various versions of CGMs are developed, because of the diffusion of glucose from the blood to the interstitial fluid, these devices lag 5 to 15 min behind blood sugar levels. Another limitation is that the implantation procedure of CGMs is relatively invasive. Additionally, these CGMs might need to be calibrated multiple times in a day via a handheld glucometer. It has been hypothesized that calibration of CGMs using fluorescent signals across the skin might change the skin color, thickness, and hair density. Sensor instability is another issue that might result in frequent replacement of the biosensor. The long-term safety profile and biocompatibility of these devices remain unknown [77,78]. Despite the stability and sensitivity of glucose biosensors, their ability to monitor glucose in a complex matrix is a critical issue that eludes CGMs from entering the market [79].

On the other hand, both insulin resistance and β-cell dysfunction were reported to be responsible for GDM Still, little is known regarding the impact of these factors on perinatal outcomes [80]. Recently, much effort has been made to isolate and protect transplanted β-cells from the immune system and preserve their function. These include several conformal coating procedures applied to islets to create nano-thin coatings, such as the chemical reaction of polymers, formation of polyion complex, and layer-by-layer polymer deposition [81,82,83,84,85,86,87,88,89,90,91]. These approaches allow adequate diffusion of glucose, nutrients, and oxygen [84]. Nevertheless, the lack of encapsulating materials that can avoid foreign body responses to implanted biomaterials while escaping host recognition are the main barriers of islet encapsulation [85].

## 5. Conclusions and Outlook

GDM is a frequent condition during pregnancy, and constant blood glucose level surveillance is required for the mother’s and baby’s health. Non-compliance, largely owing to the unpleasant side effects of standard drugs, is the primary cause of diabetes treatment failure. Moreover, there are problems in diagnosing and classifying hyperglycemia in GDM patients. To make an ideal sensing system with high-performance and easy operation, different studies focused on nanotechnology-based approaches. Recent discoveries demonstrated the advantages of using NPs as an alternative treatment for DM, using different nanomaterials, including Cu NPs, ZnO NPs, MgO NPs, CeO_2_ NPs, and Se NPs, which proved to have antidiabetic activity. Using these NPs reduces possible damages to the kidneys, pancreas, liver, and reproduction system by ameliorating oxidative stress, increasing antioxidants levels, and enhancing insulin sensitivity. Although using these nanotechnology-based approaches hold significant substantial potential for improving the care of GDM patients, one major obstacle involves the prolonged log times to elevated blood sugar levels. Restricting delivery of teratogenic drugs to the maternal compartment (such as warfarin) may reduce risks to the fetus. Alternatively, targeted delivery of drugs or nanosensors to the fetus (such as those to treat fetal arrhythmias) may minimize side effects for the mother.

Nanostructures and their respective nanocomposites—thanks to their small sizes, great biocompatibility, slow-release, and unique physicochemical characteristics—offer an appropriate means of transporting drugs, organic molecules, small molecules, and biomacromolecules to diseased cells, along with other miscellaneous applications. Presently, there are multiple nanostructures at different states of preclinical development for GDM management. Still, characterization of their systemic performance is necessary to advance nanomedicine. Interestingly, each multimodal nanostructure is unique and should be studied individually to discover how it behaves and interacts in biological systems. Moreover, investigating the pharmacokinetics, in vivo and in vitro toxicity, and efficacy of these nanostructures must be conducted before entering clinical trials. Further technological advancements are required to improve β-cell encapsulation or designing next-generation biosensors to treat and diagnose GDM.

## Data Availability

Not Applicable.
